# Living microorganisms change the information (Shannon) content of a geophysical system

**DOI:** 10.1038/s41598-017-03479-1

**Published:** 2017-06-12

**Authors:** Fiona H. M. Tang, Federico Maggi

**Affiliations:** Laboratory for Advanced Environmental Engineering Research, School of Civil Engineering, The University of Sydney, Bld. J05, 2006 Sydney, NSW Australia

## Abstract

The detection of microbial colonization in geophysical systems is becoming of interest in various disciplines of Earth and planetary sciences, including microbial ecology, biogeochemistry, geomicrobiology, and astrobiology. Microorganisms are often observed to colonize mineral surfaces, modify the reactivity of minerals either through the attachment of their own biomass or the glueing of mineral particles with their mucilaginous metabolites, and alter both the physical and chemical components of a geophysical system. Here, we hypothesise that microorganisms engineer their habitat, causing a substantial change to the information content embedded in geophysical measures (e.g., particle size and space-filling capacity). After proving this hypothesis, we introduce and test a systematic method that exploits this change in information content to detect microbial colonization in geophysical systems. Effectiveness and robustness of this method are tested using a mineral sediment suspension as a model geophysical system; tests are carried out against 105 experiments conducted with different suspension types (i.e., pure mineral and microbially-colonized) subject to different abiotic conditions, including various nutrient and mineral concentrations, and different background entropy production rates. Results reveal that this method can systematically detect microbial colonization with less than 10% error in geophysical systems with low-entropy background production rate.

## Introduction

Microorganisms have always been found to inhabit most of the natural geophysical systems on Earth even in extreme environmental conditions (e.g., hyperthermophile, piezophile, alkaliphile, and acidophile^1^). In an analogy where humans can modify the structure and functioning of ecosystems on Earth^2–4^, microorganisms play the role as ecosystem engineers because they can modify the chemistry and physical structure of their habitat^5^. Microorganisms, when assimilating, degrading, and transforming nutrients, alter the chemical composition and properties of their habitat (e.g., pH and dissolved oxygen content)^6, 7^; during these metabolic processes, microorganisms also exude organic compounds (e.g., extracellular polymeric substances)^8^ that can affect mineral surfaces^5, 9^. For example, the formation of microphytic and microbiotic soil crusts in desert and semi-desert ecosystems^10, 11^, and the formation of marine snows^12, 13^ are the results of engineering mediated by microorganisms. Studies have shown that the characteristics of soil crusts and sediment formed through microbiological activity are very different from those formed by physical processes^6, 14, 15^. These observations led us to hypothesise that microorganisms, when engineering their habitat, can also change the information content and orderliness of a geophysical system to a state different from its abiotic state.

Abiotic processes such as weathering, erosion, and chemical degradation can alter the information content of a geophysical system; the amount of information that can be added or removed by biological processes may, however, be greater than that of abiotic processes. In fact, Lovelock and Kaplan^16^ found that a planet with life (i.e., the Earth) has significantly higher statistical entropy (i.e., lower information content) in the chemical composition of its atmosphere as compared to a sterile Earth. This special ability of organisms to substantially change the information content of their habitat has been exploited by Lovelock^17, 18^ as a way to remotely detect biospheres on planets. Although Lovelock^16, 17^ suggests the possibility of using information embedded in a system to detect biological activity, this life detection theory has not yet been tested and validated to its entirety. For example, it has not yet been systematically tested if measures or quantifiers of geophysical characteristics can provide sufficient information for life detection; or, if background entropy production and other abiotic factors (e.g., environmental temperature and chemical composition) can constrain the capability of life to modify the information content of the habitat.

In this communication, we aim to examine the extent to which microbiological processes can change the information content of a geophysical system. We then revisited and extended the Lovelock’s life detection theory to establish a standard and systematic procedure that exploits the information content of geophysical characteristics to detect microbial colonization. Here, we used mineral sediment suspension as a model geophysical system to test our approach; and hence, we used the particle size and capacity (fractal) dimension as the geophysical characteristics of the system. The effectiveness and robustness of the proposed approach was tested and validated against 105 experiments conducted with different sediment suspension types (e.g., pure mineral suspensions, nutrient-affected mineral suspensions, and nutrient-affected and microbially-colonized suspensions) in waters subject to different mineral and nutrient concentrations, and different background entropy production rates.

## Methods

### Shannon information entropy

We quantify the information content embedded in a geophysical system using the Shannon information entropy *S*(*Q*|*P*)^19^, which is defined as

1 $$S(Q|P)=-{k}_{S}\sum _{i=1}^{N}{p}_{i}\,\mathrm{ln}\,{p}_{i},$$

where *Q* is a well-defined question, *P* is the knowledge about *Q* (in mathematical term, *P* is the probability distribution of the possible answers to *Q*), *N* is the number of possible answers to *Q*, *p*_*i*_ is the probability assigned to each answer *i*, and *k*_*S*_ is an arbitrary dimensional constant. We clarify here that an increase in Shannon entropy of Equation (1) signifies an increase in uncertainty and a decrease in information.

Here, we used particle size *L* and capacity dimension *d*_0_ as the model geophysical characteristics. The capacity dimension *d*_0_ is a volume fractal dimension that measures the space-filling capacity of a three-dimensional body^20, 21^. Note that, *d*_0_ has a bound between 1 and 3, with *d*_0_ = 1 signifying a line-like object and *d*_0_ = 3 signifying a solid Euclidean body. The Shannon entropy of particle size *S*(*Q*[*L*]|*P*[*L*]) and capacity dimension *S*(*Q*[*d*_0_]|*P*[*d*_0_]) distributions of a geophysical system were then calculated using Equation (1) with the questions *Q*[*L*] and *Q*[*d*_0_] defined here as:

*what could be the size L and capacity dimension d*_0_ *of a mineral particle randomly selected from a geophysical system?*

The knowledge *P* about *Q* is the probability distributions of *L* and *d*_0_. The number *N* of possible answers to *Q* in Equation (1) corresponds to the number of bins *N*_*b*_ of the probability distributions. Selection of *N*_*b*_ can be confusing because the uncertainty of a system could become *S*(*Q*|*P*) → ∞ when *N*_*b*_ → ∞, provided that there exists an infinite number of observations *n*_*o*_ with infinite precision *δ*_*o*_. In experiments where *n*_*o*_ and *δ*_*o*_ are finite, *S*(*Q*|*P*) increases to an asymptotic value where further increase in *N*_*b*_ will no longer lead to any increase in *S*(*Q*|*P*). Because this asymptotic value is generally limited by *δ*_*o*_ for samples with sufficiently large *n*_*o*_, the best choice is to calculate the probability distributions with bin width Δ*x* = *δ*_*o*_, hence, resulting in *N* = *N*_*b*_ = (*x*_*max*_ − *x*_*min*_)/Δ*x*, with *x*_*max*_ and *x*_*min*_ the maximum and minimum values of a set of observations, respectively. Note also that, *p*_*i*_ln*p*_*i*_ = 0 when *p*_*i*_ = 0 is assigned to an answer *i* (i.e., ${\mathrm{lim}}_{x\to {0}^{+}}x\,\mathrm{ln}\,x=0$ according to L’Hôpital’s rule) and *k*_*S*_ = 1 is used throughout this work (i.e., *S*(*Q*|*P*) is dimensionless).

### Microbial colonization and Biomass Index

From experimental observations (later presented in the Results), biomass-affected systems were found to have significantly higher *S*(*Q*|*P*) than biomass-free systems. Based on those observations, we defined the Biomass Index *BI* to practically detect microbial colonization in a geophysical system as,

2 $$BI(Q|{P}_{test})=\frac{S(Q|{P}_{ref})-S(Q|{P}_{test})}{|S(Q|{P}_{ref})-S(Q|{P}_{uni})|},$$

where *S*(*Q*|*P*_*ref*_), *S*(*Q*|*P*_*test*_), and *S*(*Q*|*P*_*uni*_) are the Shannon entropies of a question *Q* given the reference, test, and uniform probability distributions, respectively. Note that, *S*(*Q*|*P*_*uni*_) = *k*_*S*_ln*N* gives the maximum entropy of *Q*, implying that there is no information about *Q*. Note also that the numerator in Equation (2) is the information content of a message defined as in Shannon (1948)^19^.

According to Equation (2), if *P*_*test*_ has the same information content as *P*_*ref*_, then *BI*(*Q*|*P*_*test*_) = 0; if *P*_*test*_ has lower uncertainty (higher information) than *P*_*ref*_, then *BI*(*Q*|*P*_*test*_) > 0; if *P*_*test*_ has higher uncertainty (lower information) than *P*_*ref*_, then *BI*(*Q*|*P*_*test*_) < 0; and, if *P*_*test*_ contains no information about *Q*, then *BI*(*Q*|*P*_*test*_) = −1 (Table 1). Finally, *BI*(*Q*|*P*_*test*_) is bounded between

$$\frac{S(Q|{P}_{ref})}{|S(Q|{P}_{ref})-S(Q|{P}_{uni})|}\le BI(Q|{P}_{test})\le -1.$$

**Table 1:** Summary of implications on Biomass Index *BI* used for detecting microbially-colonized geophysical systems.

	Case 1	Case 2	Case 3	Case 4
**If**	*S*(*Q*|*Ptest*) = *S*(*Q*|*Pref*)	*S*(*Q*|*Ptest*) < *S*(*Q*|*Pref*)	*S*(*Q*|*Ptest*) > *S*(*Q*|*Pref*)	*S*(*Q*|*Ptest*) = *S*(*Q*|*Puni*)
**then**	*BI*(*Q*|*Ptest*) = 0	*BI*(*Q*|*Ptest*) > 0	*BI*(*Q*|*Ptest*) < 0	*BI*(*Q*|*Ptest*) = −1
**then**	Biomass-free	Biomass-free	Biomass-affected	Biomass-affected

The reference probability distribution *P*_*ref*_ in Equation (2) gives the “threshold entropy” that distinguishes a biomass-affected system from a biomass-free system; hence, choosing a suitable *P*_*ref*_ is important to ensure that *BI*(*Q*|*P*_*test*_) is not biased. Because the increase in *S*(*Q*|*P*) caused by biological activity is generally greater than that permitted by abiotic processes, and because *S*(*Q*|*P*) of a biomass-free system would not exceed the maximum entropy permitted by a system of its same kind, a good choice for *P*_*ref*_ would be a distribution that can provide the maximum possible entropy of a biomass-free geophysical system. In a geophysical system where the distributions have a finite mean and variance, a Gaussian distribution gives the highest possible entropy of that system. Hence, we proposed here to take the theoretical Gaussian probability distribution of a biomass-free geophysical system as ${P}_{ref}(x)=\frac{1}{\sqrt{2\pi {\sigma }^{2}}}\exp (\frac{-{(x-\mu )}^{2}}{2{\sigma }^{2}}){\rm{\Delta }}x$, with *μ* and *σ* the finite mean and standard deviation of biomass-free observations, respectively. A biomass-free test system would therefore have entropy lower than or equal to *S*(*Q*|*P*_*ref*_) and a biomass-affected test system would have entropy greater than *S*(*Q*|*P*_*ref*_).

A test system is therefore said to be biomass-affected if

$$BI(Q|{P}_{test}) < \mathrm{0,}$$

and biomass-free if

$$BI(Q|{P}_{test})\ge 0.$$

The effectiveness of *BI*(*Q*|*P*_*test*_) in detecting a microbially-colonized system is evaluated by calculating the likelihood to successfully detect a biomass-free system Λ_*M*_ = *n*_*S*_/*n*_*M*_ and the likelihood to successfully detect a biomass-affected system Λ_*B*_ = *n*_*S*_/*n*_*B*_, where *n*_*S*_ is the number of successful test, while *n*_*M*_ and *n*_*B*_ are the total number of tests for biomass-free and biomass-affected systems, respectively.

### The model geophysical system

In this work, we used mineral sediment suspension as a model geophysical system to test the approach described above. Experiments were conducted for three types of suspended particulate matter (SPM): (i) nutrient- and biomass-free (NFBF, pure mineral suspension); (ii) nutrient-affected and biomass-free (NABF, mineral suspension); and (iii) nutrient- and biomass-affected (NABA, microbially-colonized suspension).

All suspensions were prepared using Q38 kaolinite that has primary particle size between 0.6 *μ*m and 38 *μ*m with a median at 3.4 *μ*m and an average at 5.3 *μ*m, while ammonium nitrate NH_4_NO_3_ was added only to NABF and NABA suspensions. To create biomass-affected suspensions, NABA suspensions were inoculated with natural sedimentary microbial strains (uncharacterized) sampled from the Blackwattle Bay, Sydney, NSW, Australia. Note that, natural sedimentary microorganisms have a typical size of 0.3 *μ*m to 3 *μ*m^22^. NABA suspensions were incubated with the addition of glucose C_6_H_12_O_6_ at 21 °C for 21 days. The probability distributions of SPM size *L* and capacity dimension *d*_0_ were acquired by testing the prepared suspensions in a settling column equipped with a turbulence generating system, a water quality monitoring system, and a *μ*PIV system for acquiring micro-images of SPM, which have a pixel size of 1.82 *μ*m × 1.84 *μ*m. Detailed descriptions of the experimental facility, sample preparation, and experiment protocols were reported in Tang and Maggi (2016)^15^.

SPM size *L* and capacity dimension *d*_0_ were calculated from SPM micro-images processed based on the algorithm reported in Tang and Maggi (2016)^15^. *L* was calculated as the length of the minimum square enveloping a SPM micro-image^21^, whereas, *d*_0_ was calculated according to the light intensity spectral (LIS) method in Tang and Maggi (2015)^23^.

To test if abiotic factors can affect the effectiveness of *BI*(*Q*|*P*_*test*_), all the three SPM types were tested in waters with different mineral concentrations (i.e., *C*_*K*_ = {0.1, 0.2, 0.4} g/L), nutrient concentrations (i.e., [NH_4_NO_3_] = {1.5, 3.0, 6.0} mM), and turbulence shear rates (i.e., *G* = {32, 48, 64, 80, 96} s^−1^). Turbulence was used here to control the background entropy production rate^24–26^. In total, 105 sets of experiments (i.e., 15 for NFBF, 45 for NABF, and 45 for NABA) were conducted with 154, 240 SPM aggregates acquired for the analyses.

## Results

### Shannon entropy of size and capacity dimension distributions

**Figure 1:**
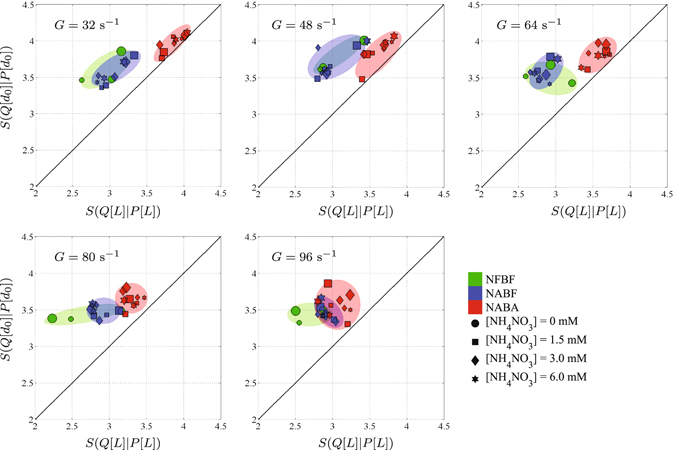
Entropy of SPM capacity dimension distribution *S*(*Q*[*d*_0_]|*P*[*d*_0_]) against entropy of SPM size distribution *S*(*Q*[*L*]|*P*[*L*]) for nutrient- and biomass-free (NFBF), nutrient-affected and biomass-free (NABF), and nutrient- and biomass-affected (NABA) suspensions with [NH_4_NO_3_] = {0, 1.5, 3.0, 6.0} mM, mineral concentration *C*_*K*_ = {0.1, 0.2, 0.4} g/L (corresponding to small, medium, and large markers, respectively), and turbulence shear rate *G* = {32, 48, 64, 80, 96} s^−1^.

Two general trends were observed in the Shannon entropy *S*(*Q*|*P*) in Fig. 1. On the one hand, the Shannon entropy of SPM capacity dimension distributions *S*(*Q*[*d*_0_]|*P*[*d*_0_]) was generally higher than size distributions *S*(*Q*[*L*]|*P*[*L*]) for all tested suspensions. On the other hand, SPM in biomass-affected suspensions (NABA) at *G* ≤ 64 s^−1^ had significantly higher *S*(*Q*[*L*]|*P*[*L*]) than biomass-free SPM (NFBF and NABF) regardless of *C*_*K*_ and [NH_4_NO_3_].

We noted that abiotic factors (*G*, *C*_*K*_, and [NH_4_NO_3_]) can cause variations in *S*(*Q*[*L*]|*P*[*L*]) and *S*(*Q*[*d*_0_]|*P*[*d*_0_]) of biomass-free suspensions; however, ANOVA analyses showed that these variations were not significant (not shown here). Conversely, *S*(*Q*[*L*]|*P*[*L*]) and *S*(*Q*[*d*_0_]|*P*[*d*_0_]) of biomass-affected suspensions decreased significantly with increasing *G* (ANOVA results not shown here). Note that biomass-affected suspensions had *S*(*Q*[*L*]|*P*[*L*]) and *S*(*Q*[*d*_0_]|*P*[*d*_0_]) relatively similar to those of biomass-free suspensions at *G* > 64 s^−1^, implying that the capacity for microorganisms to increase the entropy of a geophysical system had been constrained by high turbulence energy dissipation rates.

*S*(*Q*[*d*_0_]|*P*[*d*_0_]) was observed to be always greater than *S*(*Q*[*L*]|*P*[*L*]) because SPM aggregates of the same size can have different internal structures and space filling capacities due to different aggregation mechanisms. For example, edge-to-face and edge-to-edge aggregations can result in loosely-packed SPM aggregates with low *d*_0_, while, face-to-face aggregation can lead to densely-packed aggregates with high *d*_0_^27^. This may, therefore, suggest that the uncertainty associated in the estimation of *d*_0_ is generally higher than that of *L*.

In biomass-free geophysical systems, SPM aggregation is commonly governed by the interactions between van der Waals attraction and double-layer electrostatic repulsion forces^27^. These physico-chemical interactions will eventually bring the system to a *far-from-equilibrium* steady state where there exists a limit to the number of particles that can be withheld together by these forces^28^; in this experiment, the *far-from-equilibrium* steady state was predominantly sustained by turbulence dissipation energy. In a microbially-colonized geophysical system, mucilaginous organic matter produced by microorganisms during their metabolism increases the SPM adhesiveness and controls SPM aggregation in addition to the van der Waals attraction and electrostatic repulsion forces^29, 30^. This may then increase the number of particles that can be kept together and the number of ways particles can be structured into an aggregate. Hence, microbial activity can shift the steady state of the system away from that of biomass-free. We indeed observed that microbial activity introduced higher uncertainties (lower information) to the size and space-filling capacity of the biomass-affected SPM system (i.e., higher *S*(*Q*[*L*]|*P*[*L*]) and *S*(*Q*[*d*_0_]|*P*[*d*_0_])) as compared to that of biomass-free at *G* ≤ 64 s^−1^.

### Detecting microbial colonization in geophysical systems

After observing that microorganisms can significantly increase the Shannon entropy of a system (Fig. 1), we proposed the *BI*(*Q*|*P*_*test*_) in Equation (2) to identify if a geophysical system is microbially-colonized. Because abiotic factors were observed to cause changes in *S*(*Q*[*L*]|*P*[*L*]) and *S*(*Q*[*d*_0_]|*P*[*d*_0_]) of biomass-free suspensions, we tested if *BI*(*Q*|*P*_*test*_) is sensitive to the choice of *P*_*ref*_ and if all biomass-free suspensions would satisfy the condition *BI*(*Q*|*P*_*test*_) ≥ 0 regardless of abiotic factors. Hence, a test distribution *P*_*test*_ of either biomass-free or biomass-affected SPM was tested against 60 different *P*_*ref*_(*G*,[NH_4_NO_3_], *C*_*K*_), resulting in a total of 6300 tests.

**Figure 2:**
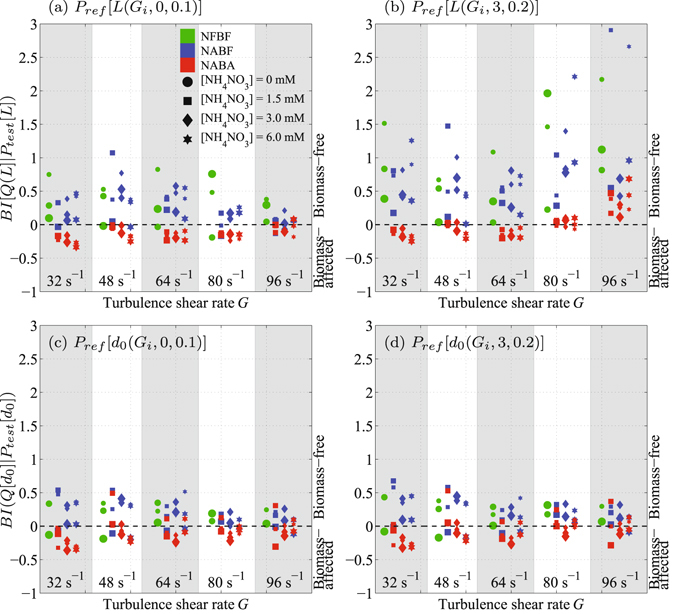
Detection of microbial colonization using SPM size *BI*(*Q*[*L*]|*P*_*test*_[*L*]) (first row) and capacity dimension distributions *BI*(*Q*[*d*_0_]|*P*_*test*_[*d*_0_]) (second row). NFBF, NABF, and NABA indicate nutrient- and biomass-free SPM, nutrient-affected and biomass-free SPM, and nutrient- and biomass-affected SPM, respectively. Panels (a and c) show detection with size *P*_*ref*_[*L*(*G*_*i*_, 0, 0.1)] and capacity dimension *P*_*ref*_[*d*_0_(*G*_*i*_, 0, 0.1)] reference distributions calculated from average *μ* and standard deviation *σ* of NFBF samples ([NH_4_NO_3_] = 0 mM) with *C*_*K*_ = 0.1 g/L at each corresponding turbulence shear rate *G*, respectively. Panels b and d show detection with *P*_*ref*_[*L*(*G*_*i*_, 3, 0.2)] and *P*_*ref*_[*d*_0_(*G*_*i*_, 3, 0.2)] calculated from *μ* and *σ* of NABF samples with [NH_4_NO_3_] = 3 mM and *C*_*K*_ = 0.2 g/L at each corresponding *G*. Small, medium, and large markers correspond to *C*_*K*_ = {0.1, 0.2, 0.4} g/L, respectively.

Figures 2a,b show that *BI*(*Q*[*L*]|*P*_*test*_[*L*]) can separate biomass-free (i.e., *BI*(*Q*|*P*_*test*_) ≥ 0) and biomass-affected (i.e., *BI*(*Q*|*P*_*test*_) < 0) SPM systems relatively well, while, *BI*(*Q*[*d*_0_]|*P*_*test*_[*d*_0_]) can do so to a lesser extent (Fig. 2c,d).

**Figure 3:**
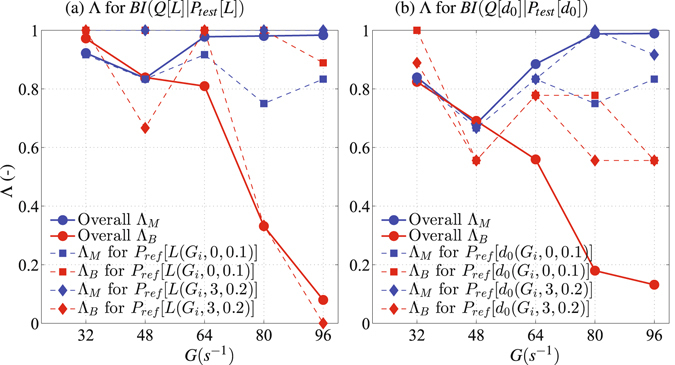
The likelihood to successfully detect a biomass-free suspension Λ_*M*_ and a biomass-affected suspension Λ_*B*_ based on (**a**) *BI*(*Q*[*L*]|*P*_*test*_[*L*]) and (**b**) *BI*(*Q*[*d*_0_]|*P*_*test*_[*d*_0_]).

Figure 3 shows that *BI*(*Q*[*L*]|*P*_*test*_[*L*]) can successfully detect biomass-free and biomass-affected systems with an overall success Λ_*M*_ and Λ_*B*_ ≥ 0.8 at *G* ≤ 64 s^−1^ (Fig. 3a); Λ_*B*_, however, decreased with increasing *G*. *BI*(*Q*[*d*_0_]|*P*_*test*_[*d*_0_]) was successful in detecting microbial colonization with Λ_*B*_ ≥ 0.8 only at *G* = 32 s^−1^ (Fig. 3b). Similar to *BI*(*Q*[*L*]|*P*_*test*_[*L*]), Λ_*B*_ of *BI*(*Q*[*d*_0_]|*P*_*test*_[*d*_0_]) decreased with increasing *G*. The successfulness in detecting biomass-free systems using either *BI*(*Q*[*L*]|*P*_*test*_[*L*]) or *BI*(*Q*[*d*_0_]|*P*_*test*_[*d*_0_]) always maintained at Λ_*M*_ ≥ 0.7 regardless of *G*.

We observed also that Λ_*M*_ was relatively insensitive to the choice of *P*_*ref*_ at all *G* values, while, Λ_*B*_ was not affected by the choice of *P*_*ref*_ only at low *G* values. For example, *BI*(*Q*[*L*]|*P*_*test*_[*L*]) consistently identified biomass-affected systems at *G* = 32 s^−1^ (i.e., Λ_*B*_ ≥ 0.97) regardless of the choice of *P*_*ref*_. The choice of *P*_*ref*_ was observed to significantly affect the resulting Λ_*B*_ at high *G* values. For example, *BI*(*Q*[*L*]|*P*_*test*_[*L*]) detected 90% of biomass-affected systems at *G* = 96 s^−1^ using *P*_*ref*_[*L*(96, 0, 0.1)], while none of the biomass-affected systems was successfully identified when *P*_*ref*_[*L*(96, 3, 0.2)] was used (Fig. 3a). The effectiveness and consistency of *BI*(*Q*|*P*_*test*_) in detecting microbially-colonized geophysical systems decreased with increasing *G* because the entropy changes in biomass-affected and biomass-free systems became less distinct at high *G*, and the ability of microorganisms to increase the entropy was somewhat overcome by the high turbulence energy dissipation rate.

## Discussion

Application of the proposed framework has relied on the assumption of mono-dispersed SPM as a geophysical model system. One may argue that the addition of microorganisms can lead to a bi-dispersed SPM that could alter its information content; thus, the observed increase in Shannon entropy in biomass-affected SPM suspensions would not be a result of microbial activity, but merely a consequence of the presence of microorganisms. However, this change in information content resulting from the inoculation of microorganisms would not be detectable in our experiments due to detector precision; that is, the *μ*PIV system with a pixel size of 1.82 *μ*m × 1.84 *μ*m limited the capturing of free-living and unattached microorganisms, which size ranged between 0.3 and 3 *μ*m. Hence, we deduced that the substantial increase in Shannon entropy observed in biomass-affected SPM suspensions was a contribution of microbial growth and metabolic processes rather than a consequence of bi-dispersed particles.

Choosing a suitable reference distribution *P*_*ref*_ is important in order to effectively make use of *BI*(*Q*|*P*_*test*_) in Equation (2). One should choose a distribution that can give the maximum possible entropy to a biomass-free geophysical system as the *P*_*ref*_, depending on the knowledge available about that system. For example, if a biomass-free system has only one finite and specified property, e.g., the mean, an exponential distribution gives the maximum possible entropy to that system. In addition, one should also make sure that the minerals present in the reference biomass-free system and the test geophysical systems have relatively similar characteristics. For example, the particle size distribution of a clay suspension may not be a suitable *P*_*ref*_ for testing suspensions made of mixed sand and clay.

Our observations that microbial activity increased the information (Shannon) entropy of geophysical measures are aligned with those in Lovelock and Kaplan^16^, who found the information entropy of the atmospheric chemical composition of an inhabited planet to be higher than that of a planet not known to sustain life. Lovelock arguments after^31^ that this increase in information entropy of the habitat is somehow related to the thermodynamic entropy produced by living organisms who must export their excess internal thermodynamic entropy to the external environment^32^. However, there is currently no direct theoretical evidence to unambiguously explain that all biological activities must follow the rule of increasing the information entropy of their habitat. Specific to our experiments, the SPM geophysical system was itself open to the external environment and, hence, we cannot conclude that the observed increase in information (Shannon) entropy was caused by the thermodynamic entropy exported from the cell interior in the absence of a comprehensive analysis of the energy and information fluxes between each compartment of the system.

Microbial detection is commonly conducted through optical techniques^33^ (e.g., microscope imaging and turbidity measurement) and biomolecular technologies such as ATP bioluminescence assays^34^, lipopolysaccharide bioassays^35^, and nucleic acid amplifications^36, 37^ (e.g., polymerase chain reaction PCR, transcription mediated amplification TMA, and nucleic acid sequence-based amplification NASBA). Although these methods are precise and can detect every single microbial cell, these methods rely on the knowledge of known Earth-based microorganisms, thus, restricting the detection of new and unknown form of microbial life on extraterrestrial planets^38^. For example, the Life Marker Chip developed for the 2018 ExoMars mission uses antibody-based microassays that require the pre-decision of target molecules in order to synthesis antibodies that recognize those targeted molecules^39^. In contrast to the above methods, the *BI*(*Q*|*P*_*test*_) proposed in this study exploits the capability of microorganisms to shift the information content of geophysical measures away from that of abiotic state without having to presume the cell structure and chemical composition of the microorganisms. Hence, this method is non Earth-centric and allows the detection of microbially-colonized geophysical systems on and off Earth.

## Conclusions

We put forth a method that makes use of measurable physical characteristics to detect microbial colonization in a geophysical system by exploiting the capability of microorganisms to change the information content of their habitat. Although there is no generally accepted principle for microorganisms to always increase the information entropy of their habitat, we showed, using the particle size *L* and capacity dimension *d*_0_ of a sedimentary suspension, that the Shannon (information) entropy of the biomass-affected geophysical system was significantly increased and shifted away from the abiotic state. The biomass-free geophysical system was, however, not significantly affected by variations in abiotic factors such as background entropy production (turbulence shear rate *G*), mineral *C*_*K*_, and nutrient [NH_4_NO_3_] concentrations. Based on these observations, the Biomass Index *BI*(*Q*|*P*_*test*_) was then developed to practically exploit information entropy to detect the colonization of microorganisms in a geophysical system. We showed that *BI*(*Q*|*P*_*test*_) can effectively be used in low-entropy background production systems with a likelihood of success greater than 90%, while the capability of *BI*(*Q*|*P*_*test*_) to detect microbial activity is lower in systems with high-entropy background production rates.
